# Impact of Annotation Level on Multisequence MRI Models for Preoperative Microvascular Invasion Prediction in Hepatocellular Carcinoma

**DOI:** 10.1148/rycan.250407

**Published:** 2026-02-20

**Authors:** Yifan Pan, Rongping Ye, Jiayi Li, Yamei Liu, Zhaodi Huang, Qiuyuan Yue, Lanmei Gao, Chuan Yan, Yueming Li

**Affiliations:** ^1^Department of Radiology, the First Affiliated Hospital of Fujian Medical University, 20 Chazhong Road, Fuzhou 350005, China; ^2^Department of Radiology, National Regional Medical Center, Binhai Campus of the First Affiliated Hospital, Fujian Medical University, Fuzhou, China; ^3^School of Medical Imaging, Fujian Medical University, Fuzhou, China; ^4^MengChao Hepatobiliary Hospital of Fujian Medical University, Fuzhou, China; ^5^Fujian Cancer Hospital, Fuzhou, China; ^6^Key Laboratory of Child Development and Learning Science of Ministry of Education, School of Biological Science and Medical Engineering, Southeast University, Nanjing, China

**Keywords:** Hepatocellular Carcinoma, Microvascular Invasion, MRI, Deep Learning, Annotation Efficiency, Model Visualization

## Abstract

**Purpose:**

To evaluate the performance of deep learning models integrating multimodal data for predicting microvascular invasion (MVI) in hepatocellular carcinoma and to investigate the impact of different manual annotation methods on performance.

**Materials and Methods:**

Patients with hepatocellular carcinoma from three institutions were included in this retrospective study; postoperative histopathology served as the reference standard for MVI. Patients from center A were divided into training and internal test sets; patients from centers B and C formed the external test set. Two manual annotations (voxel-level masks, bounding boxes) were performed on MRI scans. Deep learning models were developed using multimodal data. Model performance was evaluated using the receiver operating characteristic, calibration, and decision curve analysis, with area under the receiver operating characteristic curve (AUC) differences tested by the DeLong test.

**Results:**

A total of 281 patients were included in this study (mean age, 59.05 years ± 11.92 [SD]; 238 male). Single-sequence models achieved internal test AUCs of 0.57–0.76. Multisequence models reached AUCs of 0.86 (95% CI: 0.77, 0.95) with masks and 0.83 (95% CI: 0.73, 0.94) with bounding boxes. Multimodal fusion improved performance (mask: AUC, 0.88 [95% CI: 0.80, 0.96] vs bounding box: AUC, 0.85 [95% CI: 0.75, 0.94]; *P* = .50), with external test AUCs of 0.77 (95% CI: 0.66, 0.89) and 0.76 (95% CI: 0.64, 0.88), respectively (*P* = .40). Bounding box reduced time by 53% (mask = 3.24 minutes; bounding box = 1.52 minutes;* P* < .001).

**Conclusion:**

Multimodal fusion models improved predictive performance for MVI. Bounding box annotation achieved statistically comparable overall AUC to that of voxel-level masks while improving annotation efficiency.

**Keywords:** Hepatocellular Carcinoma, Microvascular Invasion, MRI, Deep Learning, Annotation Efficiency, Model Visualization

*Supplemental material is available for this article.*

© The Author(s) 2026. Published by the Radiological Society of North America under a CC BY 4.0 license.

See also commentary by Huang and Dong in this issue.

## Graphical abstract

**Figure:**
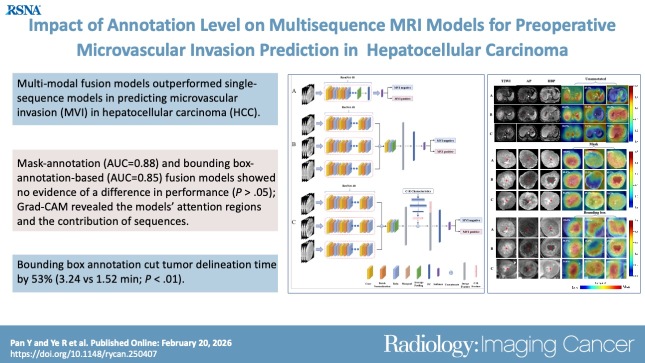

**Summary**

Bounding box annotation provides a time-efficient, clinically viable alternative to voxel-level masks for preoperative microvascular invasion prediction in hepatocellular carcinoma, offering statistically comparable performance while improving annotation efficiency.

**Key Points**

■ Multimodal fusion models (integrating multisequence MRI and clinical-radiologic features) outperformed single-sequence models in predicting microvascular invasion in hepatocellular carcinoma (single-sequence models: internal test areas under the receiver operating characteristic curve [AUCs], 0.57–0.76 [range across all sequences and annotation methods]; multimodal fusion models: internal test AUCs, 0.73 [unannotated], 0.88 [mask], and 0.85 [bounding box]; external test AUCs, 0.61 [unannotated], 0.77 [mask], and 0.76 [bounding box]).
■ We found no evidence of a difference in performance between fusion models using mask annotation (AUC, 0.88) and those using bounding box annotation (AUC, 0.85) in the internal test set (*P* = .05); notably, bounding box annotation reduced tumor delineation time by 53% (mask = 3.24 minutes; bounding box = 1.52 minutes; *P* < .001).
■ Gradient-weighted class activation mapping visualizations demonstrated that mask and bounding box annotation methods led the deep learning models to focus on tumor regions.

## Introduction

Primary liver cancer is the sixth most common cancer and the third leading cause of cancer-related deaths worldwide. Hepatocellular carcinoma (HCC) is the predominant pathology type (75%–85%), ranking sixth in global incidence and fourth in cancer-related mortality (1). Microvascular invasion (MVI) in HCC is associated with early recurrence and decreased survival rates following resection or transplantation and influences the choice of treatment modalities (2–7). Thus, accurate preoperative prediction of MVI status is essential to optimizing treatment strategies and improving prognosis (8). However, preoperative assessment of MVI remains challenging, as pathology analysis is currently the most reliable method, yet preoperative liver biopsy is invasive and limited by low accuracy, multiple uncertainties, and poor reliability (9,10).

MRI plays an indispensable role in HCC preoperative diagnosis, evaluation, and clinical decision-making (11–16). With ongoing advances in computer science, deep learning (DL) has gained substantial attention in medicine. For example, convolutional neural networks excel at learning image features and have been widely adopted. Building on convolutional neural network principles, more advanced architectures such as ResNet have demonstrated strong performance in computer vision tasks (17). Currently, multisequence MRI data have been used in several studies to develop DL models for MVI prediction (18,19). Compared with radiomics, DL demonstrates superior predictive performance, and it reduces the need for manual feature selection, thereby decreasing labor intensity and processing time (20,21).

Despite progress in DL applications for MVI prediction, to our knowledge, no standardized approach exists for tumor region annotation in imaging datasets (22,23). Accordingly, the aim of this study is to evaluate the performance of DL models integrating multimodal data for preoperative MVI prediction in HCC, to investigate the impact of different manual annotation methods on prediction accuracy, and to verify whether a time-efficient bounding box annotation strategy can achieve statistically comparable diagnostic performance to that of mask annotation.

## Materials and Methods

### Study Patients

The use of data in this retrospective study posed no harm or impact to patients. Therefore, the institutional ethics committees of the First Affiliated Hospital of Fujian Medical University, MengChao Hepatobiliary Hospital of Fujian Medical University, and Fujian Cancer Hospital, which approved this study, all waived the requirement to obtain informed consent from patients.

**Figure 1:**
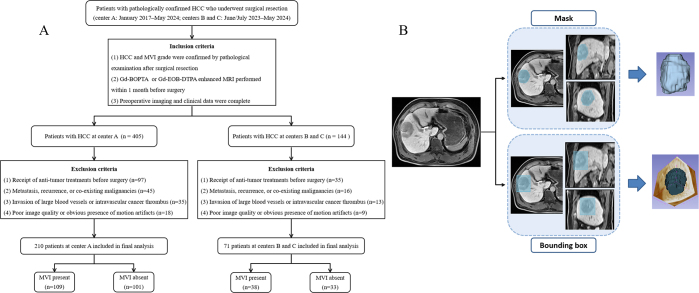
**(A)** Flowchart of patient inclusion in this study. A total of 549 patients with pathologically confirmed hepatocellular carcinoma (HCC) who underwent surgical resection were initially screened. After excluding 268 patients, 281 were included in the final analysis, with microvascular invasion (MVI) status distributions as shown. **(B)** Flowchart of mask- and bounding box–based annotation methods. Gd-BOPTA = gadobenate dimeglumine, Gd-EOB-DTPA = gadoxetate disodium.

The inclusion criteria were HCC diagnosis and MVI grade confirmed by pathology examination after surgical resection, contrast-enhanced MRI with gadobenate dimeglumine or gadoxetate disodium performed within 1 month before surgery, and complete preoperative imaging and clinical data. Exclusion criteria were receipt of preoperative antitumor treatments such as radiofrequency ablation, transhepatic arterial chemoembolization, radiation therapy, or systemic chemotherapy; presence of metastasis, recurrence, or coexisting malignancies; evidence of macrovascular invasion or intravascular tumor thrombus at imaging; and poor image quality or presence of considerable motion artifacts hindering tumor boundary assessment. Recruitment pathways and detailed inclusion and exclusion criteria are illustrated in Figure 1A.

We initially screened 549 consecutive patients with pathologically confirmed HCC who underwent surgical resection between January 2017 and May 2024 across three centers for eligibility. After excluding 268 patients because of receipt of preoperative antitumor treatments (*n* = 132); the presence of metastasis, recurrence, or coexisting malignancies (*n* = 61); evidence of macrovascular invasion or intravascular tumor thrombus at imaging (*n* = 48); and poor image quality or substantial motion artifacts hindering tumor boundary assessment (*n* = 27), we included 281 patients in the final analysis. These patients were from center A (First Affiliated Hospital of Fujian Medical University, January 2017–May 2024), center B (MengChao Hepatobiliary Hospital of Fujian Medical University, June 2023–May 2024), and center C (Fujian Cancer Hospital, July 2023–May 2024). We randomly divided patients from center A into a training set and an internal test set in a 7:3 ratio. We included patients from centers B and C in an external test set to evaluate model generalizability.

### Pathology Evaluation

MVI was defined as the presence of tumor cells within a vascular space lined by endothelium, most commonly in the portal vein, hepatic vein, or large capsular vessels of surrounding hepatic tissue, as observed under microscopy (24,25). This postoperative pathologic definition served as the reference standard for MVI assessment, and patients were classified into MVI-negative or MVI-positive groups according to this MVI criterion.

### MRI Protocol

Patients’ preoperative MRI scans were acquired in the transverse plane, including T2-weighted images; precontrast T1-weighted images; and images in the arterial phase (AP), portal venous phase, delayed phase, and hepatobiliary phase (HBP) after contrast agent (gadobenate dimeglumine, MultiHance, Bracco; gadoxetate disodium, Xian’a, Chia Tai Tianqing Pharmaceutical Group) injection (Fig S1). Detailed MRI parameters for each center are provided in Appendix S1.

### Clinical and Radiologic Characteristics

We collected preoperative clinical data, including age; sex; presence of cirrhosis or hepatitis B or C virus infection; α-fetoprotein level; platelet count; prothrombin time; and the levels of total bilirubin, albumin, γ-glutamyltransferase, alanine aminotransferase, and other relevant indicators.

All MRI scans were independently reviewed by two abdominal radiologists (R.Y. and Q.Y., with 6 and 8 years of experience, respectively), each blinded to the patients’ clinical and pathology data. In cases of disagreement, a final consensus was reached through discussion. The largest tumor was selected as the main object in patients with multiple HCCs. The radiologic characteristics assessed included the maximum tumor diameter, tumor margins, peritumoral arterial enhancement, peritumoral hypointensity on HBP, mosaic architecture, AP hyperenhancement, nonperipheral washout, tumor capsule, tumor hypointensity on HBP, intratumoral hemorrhage, intratumoral necrosis, and intratumoral steatosis.

### Tumor Annotation and Image Preprocessing

Tumor annotation and image preprocessing were performed using 3D Slicer software (version 5.0.2). N4 bias field correction was applied to normalize signal intensity values on the images. Two annotation methods were used. First, a radiologist (Y.P., with 3 years of experience) manually delineated the entire tumor across image sections, producing a volume of interest mask. Next, a three-dimensional (3D) bounding box was manually placed to enclose the tumor region, following a standardized protocol: The box was manually drawn as the smallest cuboid that fully enclosed the tumor across all sections. Priority was given to complete tumor inclusion, tolerating minimal adjacent liver parenchyma while avoiding nonhepatic structures when possible. For exophytic tumors, the bounding box was required to encompass the entire protruding lesion, even when extending beyond the liver margin. This approach balanced full tumor capture with minimization of irrelevant background, ensuring comparability across lesions. All annotation results were reviewed and modified by another radiologist (R.Y., with 6 years of experience).

The original image, mask, and bounding box were resampled to a voxel size of 1 × 1 × 1 using SimpleITK. Cropping and fusion operations were then applied to extract images containing the tumor region. Unannotated images were also included as a control group; all of the above images were resized to 64 × 64 × 16 (Fig 1B).

### Development of DL Models

**Single-sequence models**

**Figure 2:**
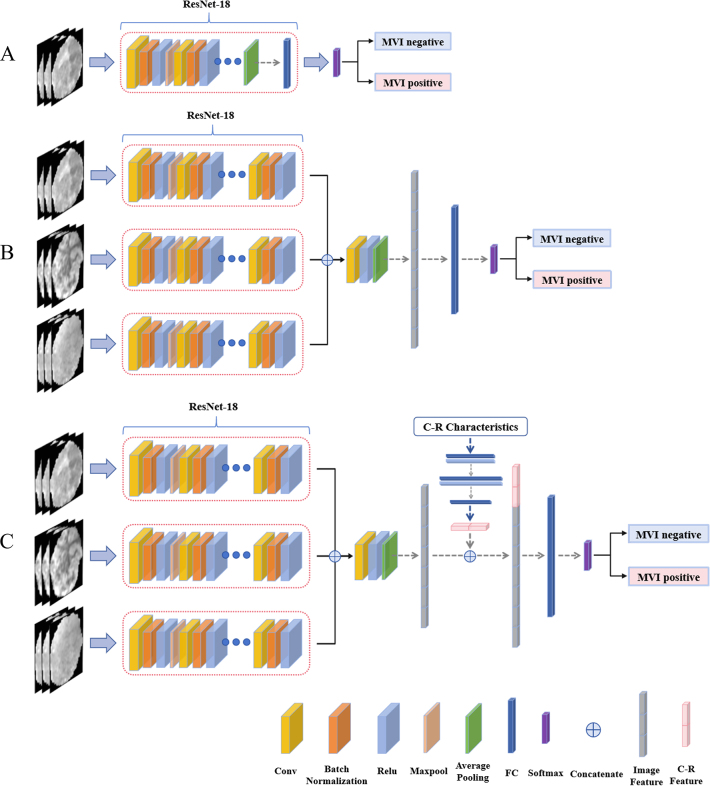
**(A)** Single-sequence model structure, **(B)** multisequence model structure, and **(C)** fusion model structure. Conv = convolution, C-R = clinical-radiologic, FC = fully connected, MVI = microvascular invasion, relu = rectified linear unit.

Using six MRI sequences, we constructed single-sequence models corresponding to different annotation levels, using ResNet-18 as the base network (Fig 2A). ResNet-18 is a 3D convolutional neural network known for its simplicity and speed, with batch normalization layers and residual connections that improve training stability and reduce overfitting.

**Multisequence models**

We developed a multisequence architecture by modifying ResNet-18 to accept three input sequences. Deep features were first extracted from the highest-performing sequences. These features were concatenated and passed through a convolutional layer. The resulting features from the global average pooling layer were then input into fully connected and SoftMax layers to generate the final prediction (Fig 2B).

### Fusion Models

We also developed a fusion architecture that integrated multisequence imaging features with clinical-radiologic variables. Following a late fusion strategy, the independent predictors identified in the multivariable analysis were converted into a one-hot encoded vector and processed through a dedicated branch of three fully connected layers. The resulting representation was then concatenated with the flattened deep image features extracted from the multisequence branch. The combined features were further processed by additional fully connected and SoftMax layers to generate the final prediction (Fig 2C). To quantify branch feature intensity, we measured the average *L*_2_ norm of the output features of each branch as an indicator of feature intensity. This choice is theoretically supported by the ability of the *L*_2_ norm to characterize the energy density of discriminative information in features (26). Notably, a larger *L*_2_ norm indicates more concentrated task-relevant information, which directly correlates with the actual contribution of a branch to the fusion task (27). During forward passes, high-dimensional tensors were extracted from the imaging branches, and low-dimensional vectors were extracted from the clinical-radiologic branch. For each sample, the *L*_2_ norm was computed across all nonbatch dimensions, and the mean and SD across the dataset were calculated to represent overall feature intensity and variability. The specific calculation formulas for each branch are as follows:


$$L_{2}norm=\sqrt{\sum_{d\in D}x_{c}^{2}}$$

Here, *D* represents the set of nonbatch dimensions, *d* is the specific dimension index in *D*, $x_{c}^{2}$ is the feature value of a branch at dimension *d*, and Σ*_d∈D_* represents the sum of all dimensions in *D*. The relative feature intensity proportion of each branch was then derived by normalizing its mean *L*_2_ norm against the total sum across all branches, which reflects the relative magnitude of feature intensity for each branch in the fusion model.

All models described above were built using the PyTorch package based on the PyCharm Community Edition 2023.3.4 platform. During training, the initial hyperparameters were set as follows: a learning rate of 0.0005, a batch size of 21, 240 training epochs, and a weight decay coefficient of 0.001. Data augmentation was applied to the training set to improve model robustness (random rotations 0°–90°, horizontal and vertical flips with a probability of 50%).

The cross-entropy loss function and the Adam optimizer were used to update model weights. The loss was calculated with the following function:


L(y,a)=−[y log a+(1−y)log(1−a)]

Here, *L*(*y,a*) represents the model loss; *y* denotes the true MVI status, and *a* is the predicted probability of being MVI positive, ranging from 0 to 1. For multisequence and fusion models involving multiple network branches, the total losses were calculated as follows:


$$L_{m}=L_{1}+L_{2}=L_{3},L_{f}=L_{1}+L_{2}+L_{3}+L_{4}.$$

Here, *L_m_* is the total loss for the multisequence model, and *L_f_* is the total loss for the fusion model. *L*_1_, *L*_2_, and *L*_3_ correspond to the losses from the image branches, while *L*_4_ represents the loss from the last branch in the fusion model. Each model underwent 10 rounds of training and validation, and we selected the model with the best performance for further analysis. A focused failure-case analysis was also conducted to explore conditions under which the bounding box fusion model underperformed compared with the mask-based model.

### Model Visualization Technology

We used the gradient-weighted class activation mapping (Grad-CAM) visualization technique to show each sequence’s contribution to prediction. All heat maps were normalized to a [0,1] range, facilitating intuitive visualization of intersequence contributions and model attention regions across three annotation levels, thereby enhancing multisequence model interpretability.

### Comparison of Annotation Methods

We also compared the time required for two annotation methods. We randomly selected 50 patients from center A. Given the clear tumor boundaries in HBP images, a radiologist annotated the tumor regions using both methods for each patient’s HBP scan. For each patient, annotation time was measured from the moment the HBP image was opened in the software until the final annotation was saved. For mask annotation, this interval covered the complete manual delineation of the tumor mask. For the bounding box method, this interval included all iterative adjustments needed to optimize the box placement while minimizing inclusion of adjacent tissue. This protocol ensured that the entire workflow for both methods was consistently timed.

### Statistical Analysis

We compared categorical variables between the MVI-positive and MVI-negative groups using the χ^2^ test; we used the Fisher exact test instead when the expected frequency of any cell in the contingency table was less than five. We compared continuous variables using the independent Student *t* test or Mann-Whitney *U* test. We conducted univariable and multivariable logistic regression analyses to evaluate the clinical and radiologic characteristics as independent variables being investigated. Variables with *P* < .10 in the univariable analysis were entered into a multivariable analysis to identify independent predictors. During this analysis, we adjusted for potential confounding variables (eg, age, sex) and calculated odds ratios with their corresponding 95% CIs. The use of a relaxed significance level (ie, *P* < .10) for variable selection is a common practice in exploratory model building to avoid prematurely excluding variables that may be significant in the multivariable context (28). The final identification of independent predictors in the multivariable model was based on a conventional threshold of *P *< .05. We determined the area under the receiver operating characteristic curve (AUC), accuracy, specificity, and sensitivity to evaluate model performance on the test sets. We performed decision curve analysis to evaluate the clinical net benefit of each model across a continuous range of threshold probabilities. Separately, the Youden index was derived from the receiver operating characteristic curve to identify the optimal cutoff value that balances sensitivity and specificity for MVI diagnosis. We used a calibration curve to evaluate the agreement between model predicted probabilities and actual outcomes. We used the DeLong test to assess differences in predictive performance between models. To compare the mean annotation times between the two methods, we used a paired *t* test—consistent with the paired design, which accounts for intrasample correlation and improves the statistical power of the comparison. Sample size was deemed adequate based on prior literature and a power calculation (power = 0.8; α = .05) to detect clinically relevant differences in annotation time. We considered findings with a two-sided *P* < .05 as statistically significant. We conducted statistical analyses using SPSS, version 27 (IBM), and Python, version 3.9 (Python Software Foundation).

## Results

### Clinical and Radiologic Characteristics

We included 281 patients with HCC (210 from center A, 71 from centers B and C; mean age, 59.05 years ± 11.92 [SD]; 238 male, 43 female). A partial summary of the baseline characteristics of patients in the training and internal test sets is shown in Table 1. In the training set, most patients had hepatitis B or C virus infection (78.9% vs 85.9%; *P* = .27) and solitary tumors (89.5% vs 90.1%; *P* = .89). Patients who were MVI positive had higher rates of α-fetoprotein of more than 400 ng/mL (35.5% vs 12.7%; *P* = .001), nonsmooth tumor margins (73.7% vs 31.0%; *P* < .001), and maximum tumor length of more than 5 cm (46.0% vs 31.0%; *P* = .06). Similarly, in the internal test set, most patients had hepatitis B or C virus infection (78.8% vs 76.7%; *P* = .84) and solitary tumors (87.9% vs 90.0%; *P* > .99); patients who were MVI positive had higher rates of α-fetoprotein of more than 400 ng/mL (42.4% vs 13.3%; *P* = .01), maximum tumor length of more than 5 cm (75.8% vs 23.3%; *P* < .001), and nonsmooth tumor margins (69.7% vs 46.7%;* P* = .06). We found no evidence of differences in any variable (*P* > .05) between the two datasets, including age (*P* = .10), sex (*P* = .15), cirrhosis (*P* = .32), maximum tumor length (*P* = .11), and α-fetoprotein levels (*P* = .54). The baseline variable details of patients with HCC from the three centers are provided in Table S1–S4. Independent risk factors for MVI identified through logistic regression analyses included nonsmooth tumor margin (odds ratio, 5.29; 95% CI: 2.19, 12.77; *P *< .001), nonperipheral washout (odds ratio, 3.90; 95% CI: 1.45, 10.49; *P *= .007), and peritumoral hypointensity on HBP (odds ratio, 3.27; 95% CI: 1.17, 9.11; *P* = .02) (Table 2). Thus, in Table 1, the distribution and comparability of baseline variables across datasets are primarily described, whereas Table 2 is focused on the association of these variables with MVI.

**Table 1:** Clinical and Radiologic Variables of Patients in the Training and Internal Test Sets

Variable	Total (*n* = 210)	Training Set (*n* = 147)		Internal Test Set (*n* = 63)		*P*_inter_
		MVI Positive (*n* = 76)	MVI Negative (*n* = 71)	MVI Positive (*n* = 33)	MVI Negative (*n* = 30)	
Clinical variable						
Sex						.15
Female	32 (15.2)	9 (11.8)	10 (14.1)	9 (27.3)	4 (13.3)	
Male	178 (84.8)	67 (88.2)	61 (85.9)	24 (72.7)	26 (86.7)	
Age (y)	59.0 ± 11.7 (15–85)	60.3 ± 11.7 (27–81)	59.8 ± 10.9 (33–85)	54.6 ± 12.3 (29–74)	58.5 ± 12.9 (15–75)	.10
Cirrhosis						.32
Absent	76 (36.2)	25 (32.9)	25 (35.2)	12 (36.7)	14 (46.7)	
Present	134 (63.8)	51 (67.1)	46 (64.8)	21 (63.6)	16 (53.3)	
HBV or HCV infection						.44
Negative	40 (19.0)	16 (21.1)	10 (14.1)	7 (21.2)	7 (23.3)	
Positive	170 (81.0)	60 (78.9)	61 (85.9)	26 (78.8)	23 (76.7)	
α-fetoprotein						.54
≤400 ng/mL	156 (74.3)	49 (64.5)	62 (87.3)	19 (57.6)	26 (86.7)	
>400 ng/mL	54 (25.7)	27 (35.5)	9 (12.7)	14 (42.4)	4 (13.3)	
Radiologic variable						
Maximum tumor length						.11
≤5 cm	121 (57.6)	41 (54.0)	49 (69.0)	8 (24.2)	23 (76.7)	
>5 cm	89 (42.4)	35 (46.0)	22 (31.0)	25 (75.8)	7 (23.3)	
Tumor margins						.45
Smooth	95 (45.2)	20 (26.3)	49 (69.0)	10 (30.3)	16 (53.3)	
Nonsmooth	115 (54.8)	56 (73.7)	22 (31.0)	23 (69.7)	14 (46.7)	
APHE						.39
Absent	33 (15.7)	17 (22.4)	4 (5.6)	10 (30.3)	2 (6.7)	
Present	177 (84.3)	59 (77.6)	67 (94.4)	23 (69.7)	28 (93.3)	
Nonperipheral washout						.70
Absent	53 (25.2)	12 (15.8)	24 (33.8)	7 (21.2)	10 (33.3)	
Present	157 (74.8)	64 (84.2)	47 (66.2)	26 (78.8)	20 (66.7)	
Tumor capsule						.36
Complete	90 (42.9)	27 (35.5)	39 (54.9)	11 (33.3)	13 (43.3)	
Absent or incomplete	120 (57.1)	49 (64.5)	32 (45.1)	22 (66.7)	17 (56.7)	
Peritumoral hypointensity on HBP						.09
Absent	144 (68.6)	44 (57.9)	62 (87.3)	15 (45.5)	23 (76.7)	
Present	66 (31.4)	32 (42.1)	9 (12.7)	18 (54.5)	7 (23.3)	

Note.—Data are numbers of patients with percentages in parentheses or means ± SDs with ranges in parentheses. The *P* value was determined with the χ^2^ test or Fisher exact test for categorical variables. *P*_inter_ is the comparison between the training and internal test sets to verify dataset consistency. APHE = arterial phase hyperenhancement, HBP = hepatobiliary phase, HBV = hepatitis B virus, HCV = hepatitis C virus, MVI = microvascular invasion.

**Table 2:** Results of Univariable Analysis and Multivariable Analysis in the Training Set

Variable	Univariable Analysis		Multivariable Analysis			
	OR	*P*_univariable_	No. of Events*	Coeff	OR	*P*_multivariable_
Sex (Female vs male)	1.22 (0.47, 3.20)	.69	…	…	…	…
Age	1.00 (0.98, 1.03)	.20	…	…	…	…
No. of tumors (solitary vs multiple)	1.08 (0.37, 3.14)	.89	…	…	…	…
Cirrhosis (absent vs present)	1.11 (0.56, 2.19)	.77	…	…	…	…
HBV or HCV infection (negative vs positive)	0.62 (0.26, 1.46)	.27	…	…	…	…
α-fetoprotein level (≤400 vs >400 ng/mL)	3.80 (1.64, 8.81)	.002	49/27	1.03	2.80 (0.99, 7.94)	.053
PLT count (≤125 vs >125 ×10^9^/L)	0.49 (0.21, 1.15)	.10	…	…	…	…
PT (≤13 vs >13 sec)	1.23 (0.65, 2.78)	.56	…	…	…	…
PT-INR (≤1.0 vs >1.0)	1.35 (0.30, 2.86)	.42	…	…	…	…
TBIL level (≤20.5 vs >20.5 μmol/L)	1.57 (0.68, 3.64)	.29	…	…	…	…
ALB level (≤40 vs >40 g/L)	1.00 (0.52, 1.94)	>.99	…	…	…	…
GGT level (≤60 vs >60 U/L)	1.47 (0.74, 2.92)	.27	…	…	…	…
ALT level (≤50 vs >50 U/L)	0.84 (0.35, 1.98)	.69	…	…	…	…
AST level (≤40 vs >40 U/L)	1.19 (0.53, 2.69)	.68	…	…	…	…
Maximum tumor length (≤5 vs >5 cm)	1.90 (0.97, 3.74)	.06	41/35	−0.41	0.67 (0.23, 1.89)	.45
Tumor margins (smooth vs nonsmooth)	6.24 (3.05, 12.77)	<.001	20/56	1.67	5.29 (2.19, 12.77)	<.001^†^
APHE (absent vs present)	0.21 (0.07, 0.65)	.007	17/59	−1.08	0.34 (0.08, 1.46)	.15
Nonperipheral washout (absent vs present)	2.72 (1.24, 5.99)	.01	12/64	1.36	3.90 (1.45, 10.49)	.007^†^
Peritumoral arterial enhancement (absent vs present)	1.53 (0.78, 3.03)	.22	…	…	…	…
Tumor capsule (complete vs absent or incomplete)	2.21 (1.14, 4.21)	.02	27/49	0.38	1.46 (0.62, 3.44)	.38
Tumor hypointensity on HBP (absent vs present)	3.31 (0.34, 32.57)	.31	…	…	…	…
Peritumoral hypointensity on HBP (absent vs present)	5.01 (2.18, 11.54)	<.001	44/32	1.18	3.27 (1.17, 9.11)	.02^†^
Mosaic architecture (absent vs present)	2.49 (1.27, 4.85)	.008	25/51	−0.30	0.74 (0.28, 1.95)	.54
Hemorrhage (absent vs present)	1.77 (0.82, 3.79)	.14	…	…	…	…
Necrosis (absent vs present)	2.82 (1.23, 6.43)	.01	52/24	0.88	2.40 (0.68, 8.50)	.18
Steatosis (absent vs present)	0.63 (0.27, 1.49)	.29	…	…	…	…

Note.—Data in parentheses are 95% CIs. Odds ratios (ORs) were derived from univariable and multivariable logistic regression analyses—binary variables reflect second category likelihood relative to the first, and continuous variables denote a one-unit increase effect. *P*_univariable_ is the *P* value of univariable logistic regression. *P*_multivariable_ is the *P* value of multivariable logistic regression. Variables with *P *< .10 in the univariable analysis or with established clinical relevance were entered into the multivariable model. ALB = albumin, ALT = alanine aminotransferase, APHE = arterial phase hyperenhancement, AST = aspartate aminotransferase, Coeff = logistic regression coefficient, GGT = γ-glutamyltransferase, HBV = hepatitis B virus, HBP = hepatobiliary phase, HCV = hepatitis C virus, PLT = platelet, PT = prothrombin time, PT-INR = prothrombin time–international normalized ratio, TBIL = total bilirubin.

* No. of events is the MVI-positive counts (X/Y = variable positive/variable negative).

^†^ Variables ultimately selected through multivariable logistic regression analysis (*P* < .05).

### Performance of Single-Sequence Models

The performance metrics of the single-sequence models are summarized in Table 3. Among models using unannotated images, masks, and bounding boxes, those based on T2-weighted, AP, and HBP sequences outperformed models based on precontrast T1-weighted, portal venous phase, and delayed phase sequences in the internal test set. The AUCs of the mask- and bounding box–based models exceeded 0.7.

**Table 3:** Performance Metrics for Models Based on Different Levels of Annotation in the Internal Test Set

Level of Annotation and Model	AUC	Accuracy	Sensitivity	Specificity
Unannotated				
T2WI	0.63 (0.49, 0.77)	70 (44/63)	85 (28/33)	47 (14/30)
Precontrast T1WI	0.58 (0.44, 0.72)	60 (38/63)	82 (27/33)	37 (11/30)
AP	0.64 (0.50, 0.78)	67 (42/63)	70 (23/33)	67 (20/30)
PVP	0.57 (0.42, 0.72)	62 (39/63)	79 (26/33)	43 (13/30)
DP	0.58 (0.44, 0.72)	62 (39/63)	85 (28/33)	37 (11/30)
HBP	0.59 (0.45, 0.74)	64 (40/63)	76 (25/33)	50 (15/30)
Multisequence	0.66 (0.53, 0.80)	67 (42/63)	76 (25/33)	57 (17/30)
Fusion	0.73 (0.60, 0.86)	71 (45/63)	88 (29/33)	53 (16/30)
Mask				
T2WI	0.72 (0.59, 0.85)	70 (44/63)	76 (25/33)	63 (19/30)
Precontrast T1WI	0.68 (0.54, 0.82)	68 (43/63)	73 (24/33)	63 (19/30)
AP	0.76 (0.64, 0.88)	76 (48/63)	70 (23/33)	83 (25/30)
PVP	0.70 (0.57, 0.83)	70 (44/63)	55 (18/33)	87 (26/30)
DP	0.69 (0.55, 0.82)	68 (43/63)	76 (25/33)	60 (18/30)
HBP	0.71 (0.58, 0.85)	70 (44/63)	67 (22/33)	73 (22/30)
Multisequence	0.86 (0.77, 0.95)	79 (50/63)	70 (23/33)	90 (27/30)
Fusion	0.88 (0.80, 0.96)	79 (50/63)	76 (25/33)	83 (25/30)
Bounding box				
T2WI	0.73 (0.60, 0.85)	70 (44/63)	58 (19/33)	83 (25/30)
Precontrast T1WI	0.69 (0.56, 0.82)	70 (44/63)	52 (17/33)	90 (27/30)
AP	0.74 (0.61, 0.87)	68 (43/63)	55 (18/33)	83 (25/30)
PVP	0.67 (0.53, 0.80)	65 (41/63)	46 (15/33)	87 (26/30)
DP	0.67 (0.53, 0.81)	65 (41/63)	49 (16/33)	83 (25/30)
HBP	0.70 (0.57, 0.83)	71 (45/63)	70 (23/33)	73 (22/30)
Multisequence	0.83 (0.73, 0.94)	76 (48/63)	64 (21/33)	90 (27/30)
Fusion	0.85 (0.75, 0.94)	78 (49/63)	91 (30/33)	63 (19/30)

Note.—For area under the receiver operating characteristic curve (AUC), data in parentheses are 95% CIs. For accuracy, sensitivity, and specificity, the data are percentages, and data in parentheses are numbers/numbers. AP = arterial phase, DP = delayed phase, HBP = hepatobiliary phase, PVP = portal venous phase, T1WI = T1-weighted imaging, T2WI = T2-weighted imaging.

### Performance of Multisequence Models

**Figure 3:**
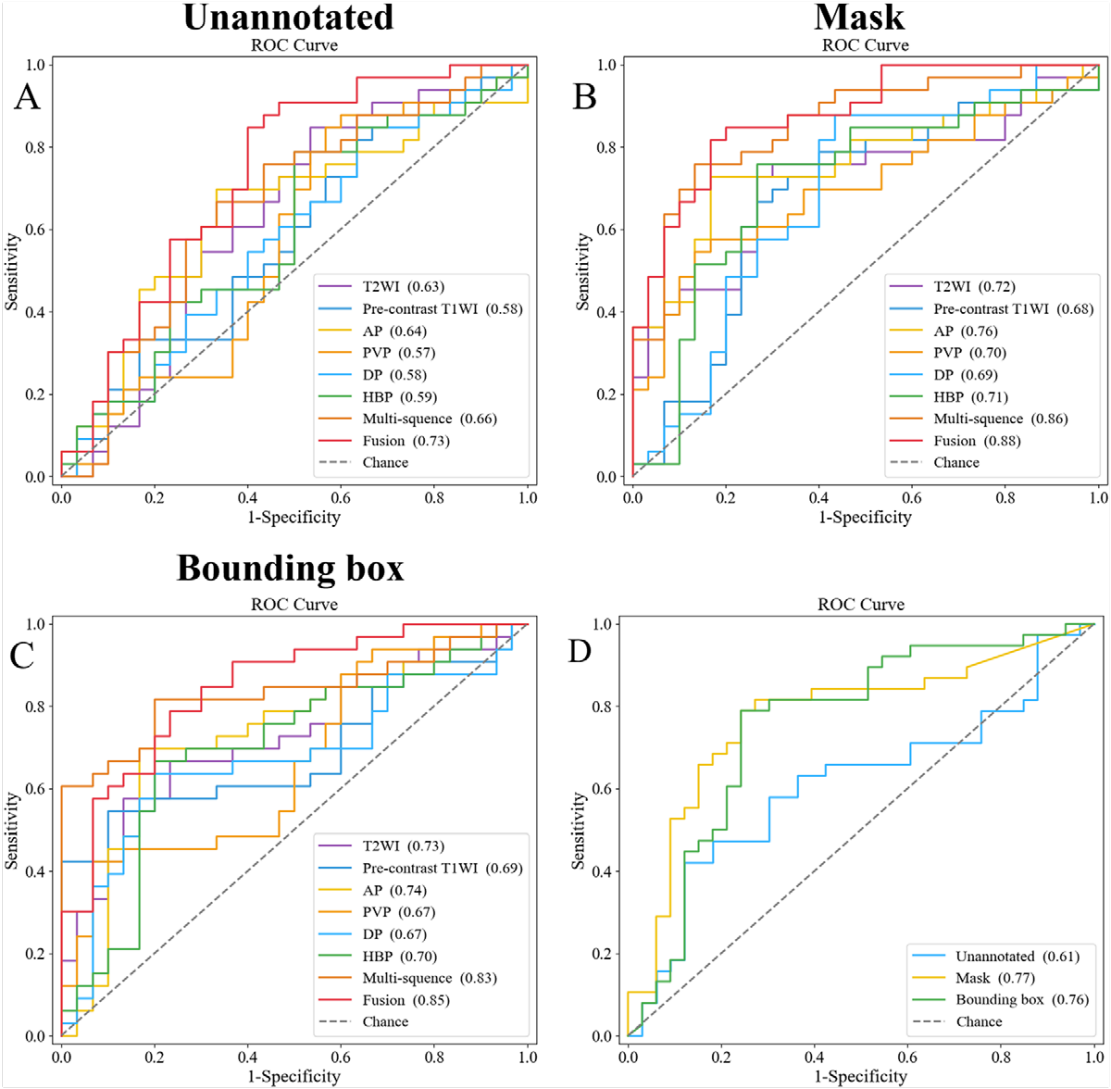
Line graphs of the receiver operating characteristic (ROC) curves of **(A)** unannotated, **(B)** mask, and **(C)** bounding box models in the internal test set and **(D)** fusion models under two annotations in the external test set. Values in parentheses are areas under the ROC curve (AUC). AP = arterial phase, DP = delayed phase, HBP = hepatobiliary phase, PVP = portal venous phase, T1WI = T1-weighted imaging, T2WI = T2-weighted imaging.

We evaluated the performance of multisequence models based on a combination of T2-weighted, AP, and HBP imaging features in the internal test set. The models based on unannotated images, masks, and bounding boxes had AUCs of 0.66 (95% CI: 0.53, 0.80), 0.86 (95% CI: 0.77, 0.95), and 0.83 (95% CI: 0.73, 0.94), respectively (Fig 3).

### Performance of Fusion Models

After integrating radiologic features, the fusion model based on unannotated images had an AUC of 0.73 (95% CI: 0.60, 0.86). The fusion models using masks and bounding boxes had AUCs that increased to 0.88 (95% CI: 0.80, 0.96) and 0.85 (95% CI: 0.75, 0.94), respectively. The accuracy, sensitivity, and specificity were 79% (50 of 63), 76% (25 of 33), and 83% (25 of 30) for the mask-based model and 78% (49 of 63), 91% (30 of 33), and 63% (19 of 30) for the bounding box–based model, respectively. The mask- and bounding box–based models in the external test set had AUCs of 0.77 (95% CI: 0.66, 0.89) and 0.76 (95% CI: 0.64, 0.88), respectively. Detailed performance metrics, including accuracy, sensitivity, and specificity, are presented in Table S5, and a comprehensive performance analysis visualization is presented in Figure S2.

To clarify the feature intensity of different data types in the fusion model, we quantified branch-specific feature intensity using the internal test set (Table S6). Across all annotation settings, HBP accounted for 41.14%–47.57% of the total intensity. AP contributed 24.45%–29.02%, and T2-weighted imaging contributed 21.97%–28.36%. The clinical-radiologic branch exhibited consistent contribution rates of 3.65%–6.05%, which aligns with the predictive value demonstrated in multivariable analysis.

### Comparison between Models

**Figure 4:**
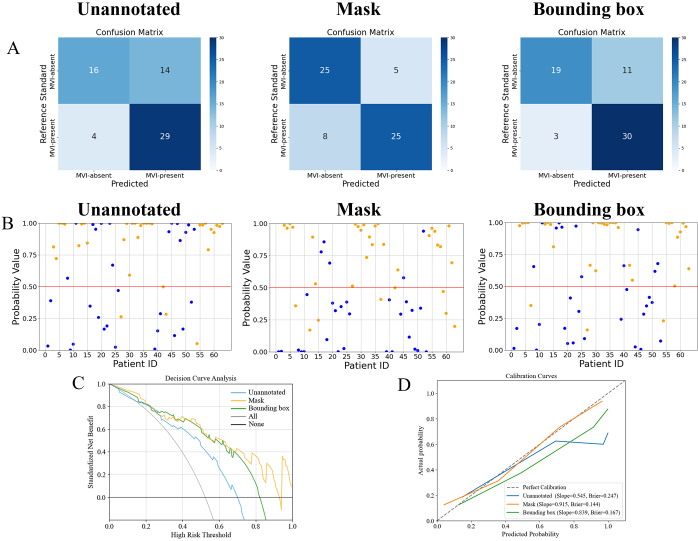
**(A)** Confusion matrix diagrams; **(B)** probability scatter distribution diagrams (blue = patient who was microvascular invasion [MVI] negative; orange = patient who was MVI positive); and **(C)** decision curves and **(D)** calibration curves of the fusion models corresponding to the unannotated, mask, and bounding box in the internal test set.

Comparative analysis of fusion model performance revealed notable differences. Specifically, analysis of the confusion matrix and probability scatterplot revealed that the mask-based model exhibited more balanced classification across negative and positive groups. In contrast, the unannotated model and bounding box–based models showed higher detection rates for patients who were MVI positive, with higher predicted probability scores (Fig 4A, 4B). Decision curve analysis results demonstrated that the mask-based model offered greater net clinical benefit, particularly in high-risk thresholds (Fig 4C). Analysis of the calibration curve revealed that models using masks and bounding boxes had similar slopes (0.915 vs 0.839) and Brier scores (0.144 vs 0.167), both outperforming the model using unannotated images (Fig 4D).

The DeLong test revealed that the fusion model based on unannotated images performed worse in both test sets (all *P *< .05), while we found no evidence of a difference in performance between mask- and bounding box–based models (*P*_internal_ = 0.50; *P*_external_ = 0.40). A full summary of all pairwise DeLong test comparisons is provided in Table S7.

### Grad-CAM Visualization Results

**Figure 5:**
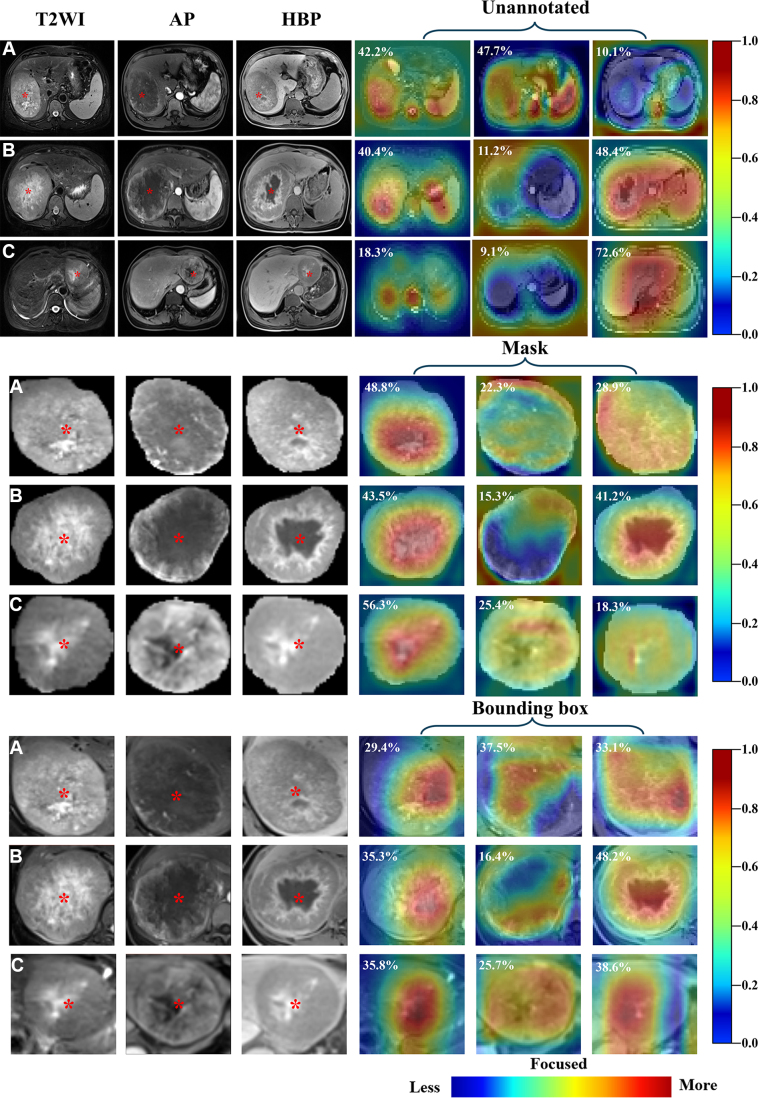
Visualization of gradient-weighted class activation mapping (Grad-CAM) heat maps for patients who were microvascular invasion (MVI) positive across three annotation methods. **(A–C)** T2-weighted images (T2WIs), arterial phase (AP) images, and hepatobiliary phase (HBP) images along with heat maps for three patients who were MVI positive; the red asterisk denotes the specific location of the tumor. The heat maps represent three input types: unannotated images, bounding box annotation, and mask annotation. Dark red areas indicate regions with the maximum contribution to the model prediction. The white numbers denote the Grad-CAM contribution proportions of each sequence.

In patients who were MVI positive, Grad-CAM heat maps revealed different attention levels across the three annotation strategies (Fig 5). Without annotation, model attention was relatively diffuse and not well confined to the tumor. Both mask- and bounding box–based models showed focused activation within the intratumoral regions, with the red areas indicating the strongest contribution to prediction. For patients who were MVI negative, representative heat maps are shown in Figure S3, where both annotation-based models displayed weak and nonfocal activation, indicating the absence of strong tumor-centered attention. Each sequence’s Grad-CAM contribution proportion are reflected in the figures, with detailed gradient contribution statistics provided in Figure S4 and Table S8.

### Annotation Method Comparison

**Figure 6:**
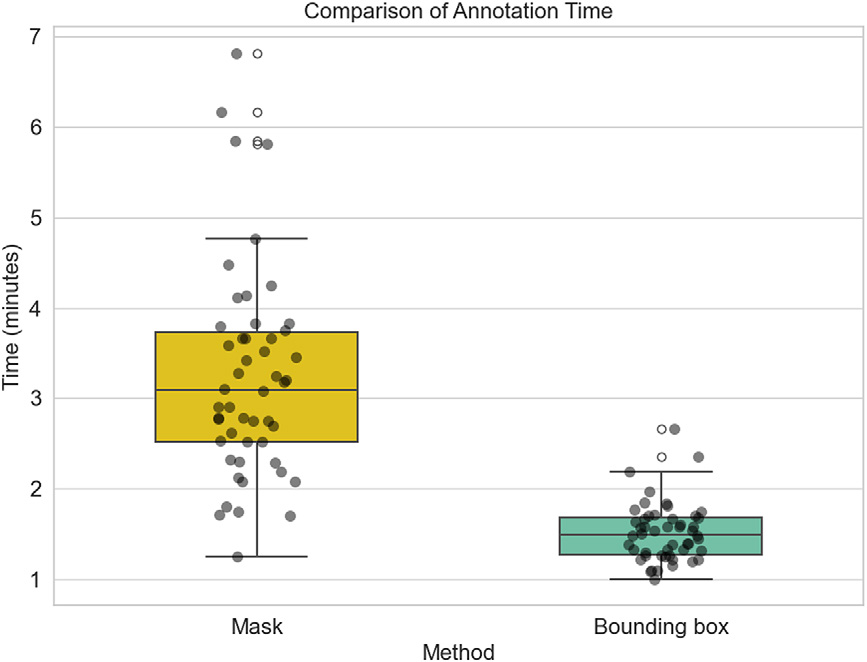
Box plot shows a comparison of the time required for mask annotation and bounding box annotation. The yellow and green boxes correspond to mask and bounding box annotation time, respectively. Each box represents the IQR (25th–75th percentiles) of times, with the internal horizontal line denoting the median. Whiskers (vertical lines) mark the farthest data points within 1.5 × IQR of the IQR bounds. Individual dots show per-sample annotation times.

In the analysis of 50 random patients from center A, 26 tumors were larger than 5 cm, whereas 24 were smaller than 5 cm. The average annotation time per patient with the mask method was 3.24 minutes, whereas this time was only 1.52 minutes with the bounding box method (Fig 6, Table S9) (*P* < .001).

### Failure-Case Analysis of the Bounding Box Model

A focused failure-case analysis was performed to explore conditions under which the bounding box fusion model underperformed compared with the mask-based model. In total, seven such cases were identified. Among these, three involved very small tumors (<2 cm), in which the bounding box inevitably encompassed a disproportionately large amount of surrounding background tissue, diluting the relevant intratumoral signal. Four additional cases featured tumors with highly irregular or spiculated margins, for which the rectangular bounding box failed to accurately capture critical boundary information, leading to loss of discriminative features (Fig S5).

## Discussion

MVI is a key risk factor for poor outcomes and recurrence in patients with HCC. DL models based on medical imaging have emerged as a promising method for predicting MVI (29). In our study, we evaluated the performance of DL models based on MRI using two manual annotation methods, with unannotated images serving as a control group. The results indicated that manual annotation significantly increased model performance by excluding tissue information unrelated to MVI. AUC reflects the model’s ability to distinguish MVI-positive from MVI-negative patients with HCC. Meanwhile, the mask-based model (AUC, 0.88 [95% CI: 0.80, 0.96] vs 0.77 [95% CI: 0.66, 0.89]) and bounding box–based model (AUC, 0.85 [95% CI: 0.75, 0.94] vs 0.76 [95% CI: 0.64, 0.88]) showed no statistical difference in predictive performance (*P* = .50 and *P *= .40, respectively).

To our knowledge, no consensus currently exists regarding the optimal image input format for MVI prediction; in some studies, two-dimensional sections were used, while in others, 3D volumetric data were used (30–32). Although 3D input may increase computational demands, we used the whole tumor volume in our study to preserve spatial tumor information as comprehensively as possible (33). DL excels at integrating multimodal data, leading to better lesion characterization than single-modality approaches (33–37). In our study, single-sequence models based on T2-weighted, AP, and HBP sequences demonstrated performance superior to that of other sequences. Each of these three sequences provides unique and complementary information about the tumor. T2-weighted offers excellent contrast for visualizing tumor boundaries and internal structure, aiding in the detection of tumor heterogeneity. The AP is essential for capturing tumor blood supply and perfusion characteristics, often linked to aggressive tumor biology and MVI. The HBP reflects tumor cell function; reduced contrast material uptake in tumors on HBP images is indicative of poorly differentiated tumor cells, which is strongly correlated with MVI. Relying on a single sequence can omit key features, which compromises model stability and accuracy. Our multisequence model integrates complementary information from T2-weighted, AP, and HBP sequences, leading to a significant performance gain over any single-sequence model. This result indicates that different imaging modalities provide complementary information essential for a more comprehensive tumor characterization (36).

Integrating clinical data with imaging features is an emerging trend in DL research. This fusion enables models to better reflect a patient’s condition and improves model variability in clinical scenarios (20). Building on this, we fused selected radiologic features with our multisequence models and observed performance improvement. Although the model using masks achieved a slightly higher AUC in both the internal and external test sets, we found no evidence of a difference in AUC between the two models in the DeLong test. The bounding box may capture more useful information, but it may also include irrelevant tissues, potentially introducing noise. Nonetheless, our findings suggest that DL models still perform well despite such interference. This finding may reflect the strong capacity of DL to automatically identify relevant features while suppressing background noise during feature extraction. Although the two annotation strategies yielded statistically comparable overall AUC, their clinical utility differs. Specifically, the mask-based strategy favored specificity, whereas the bounding box approach achieved notably higher sensitivity. This divergence likely stems from the inclusion of peritumoral tissue in bounding boxes, which provides contextual cues for detecting MVI-positive cases but may introduce background noise. This result explains its slightly lower performance in some metrics than the mask-based strategy. Nevertheless, given that missed diagnoses risk suboptimal treatment, the high sensitivity and efficiency of the bounding box strategy make it ideal for initial screening.

Recent advances in weakly supervised prompt learning and semisupervised classification suggest leveraging limited data or automatic lesion localization for annotation initialization (38–40). Such approaches merit further exploration to reduce manual annotation burden.

Currently, several studies share certain similarities with our work, but important distinctions remain. For instance, Zhang et al (41) investigated annotation style equivalence in two-dimensional semantic segmentation. Although this methodologic study provided valuable insights, it did not address predictive modeling tasks or focus on HCC. Our study extends this line of research by using 3D multisequence MRI to predict preoperative MVI in HCC, thereby providing disease-specific and clinically relevant evidence that complements prior methodologic research. Moreover, our failure-case analysis showed that bounding box annotations may be less reliable for very small or highly irregular tumors, highlighting scenarios in which precise masks remain necessary. Further analysis of bounding box error for such lesions reinforced that mask annotation offers distinct advantages for very small or highly irregular tumors. Thus, in clinical practice, clinicians may optimize annotation efficiency by using mask annotation for small or highly irregular tumors, whereas bounding box annotation can be used for the majority of tumors with normal size or regular shape. Although the predictive accuracy of our model is comparable to that of other multi-institutional studies, our central contribution lies in demonstrating that such performance can be achieved with a 53% reduction in annotation time. This efficiency gain has important implications, as it lowers the barrier for large-scale dataset construction and accelerates clinical artificial intelligence deployment. Taken together, and compared with studies such as that of Wang et al (42), who primarily focused on developing a high-performance model, our work underscores the methodologic impact of annotation strategies and provides complementary insights into the clinical translation of artificial intelligence for HCC.

We further validated the generalizability of the model using multicenter external datasets. The external test serves as a comprehensive assessment of model stability and improves model credibility. Liu et al (32) developed DL and machine learning models using patients’ AP images and clinical data, and they independently evaluated model stability in both internal and external test cohorts. We found that, compared with internal test set performance, the predictive performance of fusion models decreased in the external test set. This finding may be due to variations in data distributions—such as differences in MRI equipment, contrast agents, or scanning parameters—across centers, which can introduce deviations in image quality.

The black-box nature of DL models often limits understanding of the relationship between deep features and predictive outcomes (43). To address this, Wesp et al (44) applied the Grad-CAM++ visualization technique to interpret the predictions of the no-SEG model. Similarly, to better understand the decision-making process of our model, we reversed the model weights and generated a heat map using Grad-CAM to identify attention areas when predicting MVI. The comparative Grad-CAM analysis findings further supported the interpretability of our multisequence models. For patients who were MVI positive, both mask- and bounding box–based models consistently focused on intratumoral regions, whereas the unannotated model showed more diffuse and nonspecific activation. This finding indicates that annotation provides valuable spatial constraints that guide the model toward biologically meaningful features. In contrast, for patients who were MVI negative, both annotation-based models exhibited weak and nonfocal activation, reinforcing the specificity of tumor-centered attention in the presence of MVI-related features. Collectively, these findings highlight not only the consistency of the attention of the model across different annotation strategies but also the critical role of annotation in improving interpretability and reducing spurious activations. A key challenge with the bounding box approach is its difficulty in capturing irregularly shaped tumors. In our protocol, we prioritized making sure the entire tumor was included, even if this meant also including some surrounding nontumor tissue. Although this could introduce extra background as noise, our Grad-CAM results suggest that the model still focused on the tumor itself and was robust to the additional tissue. In contrast, leaving out part of the tumor would likely be more harmful, as important diagnostic features at the tumor edges could be missed.

To further explore the model’s multimodal feature extraction mechanism, we conducted a two-level quantitative analysis on the internal test set. First, *L*_2_ norm analysis revealed a consistent hierarchy of feature intensity across all annotation strategies, where the HBP contributed the highest proportion, followed by AP and T2-weighted imaging. This finding confirms that all models robustly encode HBP signals regardless of annotation precision. Furthermore, gradient-based analysis elucidated how these features influence decision-making. Contrary to concerns about HBP underuse, the bounding box model assigned the highest decision weight to HBP, surpassing the mask-based model. Notably, the bounding box model also demonstrated a stronger reliance on AP features compared with the mask-based mode. This distinct weighting strategy suggests that the bounding box model actively leverages hemodynamic cues from the peritumoral tissue, a context preserved by the box but excluded by masks, to enhance detection. This finding provides a mechanistic explanation for its superior diagnostic sensitivity.

Finally, we compared the time required for the two annotation methods. Results showed that the bounding box approach reduced the average annotation time from 3.24 minutes (mask annotation) to 1.52 minutes (bounding box annotation), corresponding to considerable cumulative time savings per patient in our multisequence workflow, especially when annotating large-scale datasets, which can save substantial effort and time. From a clinical perspective, bounding boxes are both practical and easily adoptable, as they require no specialized software and can be seamlessly integrated into routine radiologic workflows. Moreover, this benefit is likely underestimated, since voxel-level annotation is cognitively demanding and increasingly affected by annotator fatigue during large-volume tasks, whereas bounding box annotation is simpler and more resilient. Although voxel masks remain indispensable for downstream applications such as radiomics and surgical planning, for predictive modeling, the bounding box strategy provides an optimal balance between efficiency and accuracy, substantially lowering the barrier to large-scale dataset construction and accelerating clinical artificial intelligence development.

Our study had limitations. First, it is a retrospective study, which may introduce potential patient selection bias. Second, although the peritumoral region is known to correlate with MVI status, we did not further investigate this region in our study. Third, the model was trained and validated using data from only three centers, and further validation in a broader sample of patients with HCC from diverse institutions remains necessary. The bounding box strategy has potential limitations and requires refinement to fit diverse clinical needs; thus, prospective studies, peritumoral analysis, and exploration of emerging technologies to reduce manual annotation reliance will enable large-scale clinical deployment.

In conclusion, our findings suggest that fusing multisequence imaging with radiologic features improves model predictive accuracy. We found no evidence of a difference in performance between the two fusion models. Visualizing the decision-making process of the model provided insight into the relationship between imaging features and MVI. Accurate preoperative prediction of MVI may aid clinical decision-making, and bounding box annotations offer a time-efficient approach while maintaining statistically comparable AUC performance to voxel-level masks.

## Supplemental Files

Appendix S1, Tables S1-S9, Figures S1-S5

Conflicts of Interest
