# Supplementary material for: Impact of Annotation Level on Multisequence MRI Models for Preoperative Microvascular Invasion Prediction in Hepatocellular Carcinoma
Source: Radiol Imaging Cancer. 2026 Feb 20;8(2):e250407. doi: 10.1148/rycan.250407 (PMC13036670; doi:10.1148/rycan.250407)
Supplement: Appendix S1, Tables S1-S9, Figures S1-S5 [file rycan250407suppa1.pdf]

Supplemental materials undergo peer review but are not copyedited, and may include typographical errors.

©RSNA, 2026
10.1148/rycan.250407

Figure S1. Examples of MR imaging of HCC patients

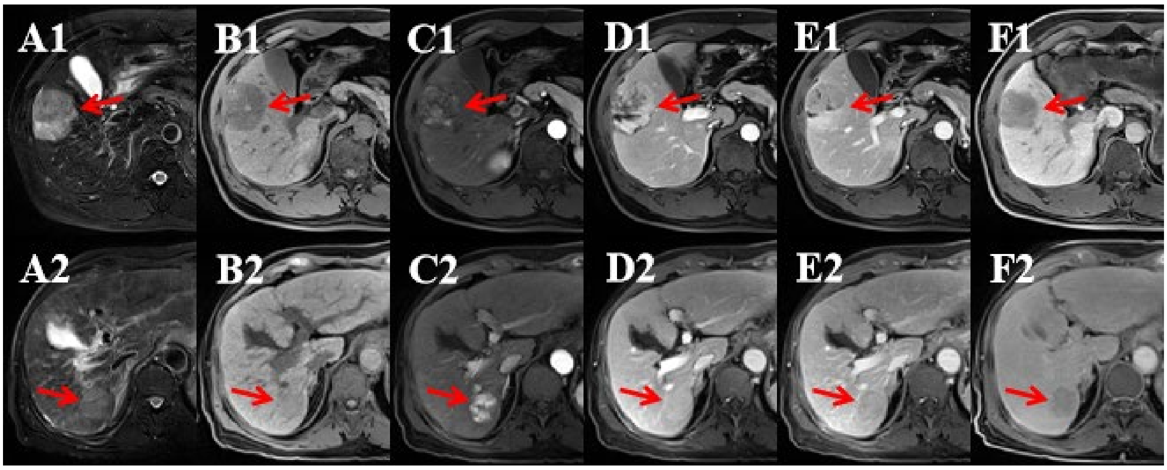

(A1-F1) Male HCC patient, 50 years old, confirmed MVI positive by postoperative pathology, (A2-F2) Female HCC patient, 69 years old, confirmed MVI negative by postoperative pathology.

Figures show the patient's T2-weighted imaging, pre-contrast T1-weighted imaging, arterial phase, portal venous phase, delayed phase, and hepatobiliary phase sequences, respectively.

Supplemental materials undergo peer review but are not copyedited, and may include typographical errors.

Figure S2. Performance of fusion models in the external test set

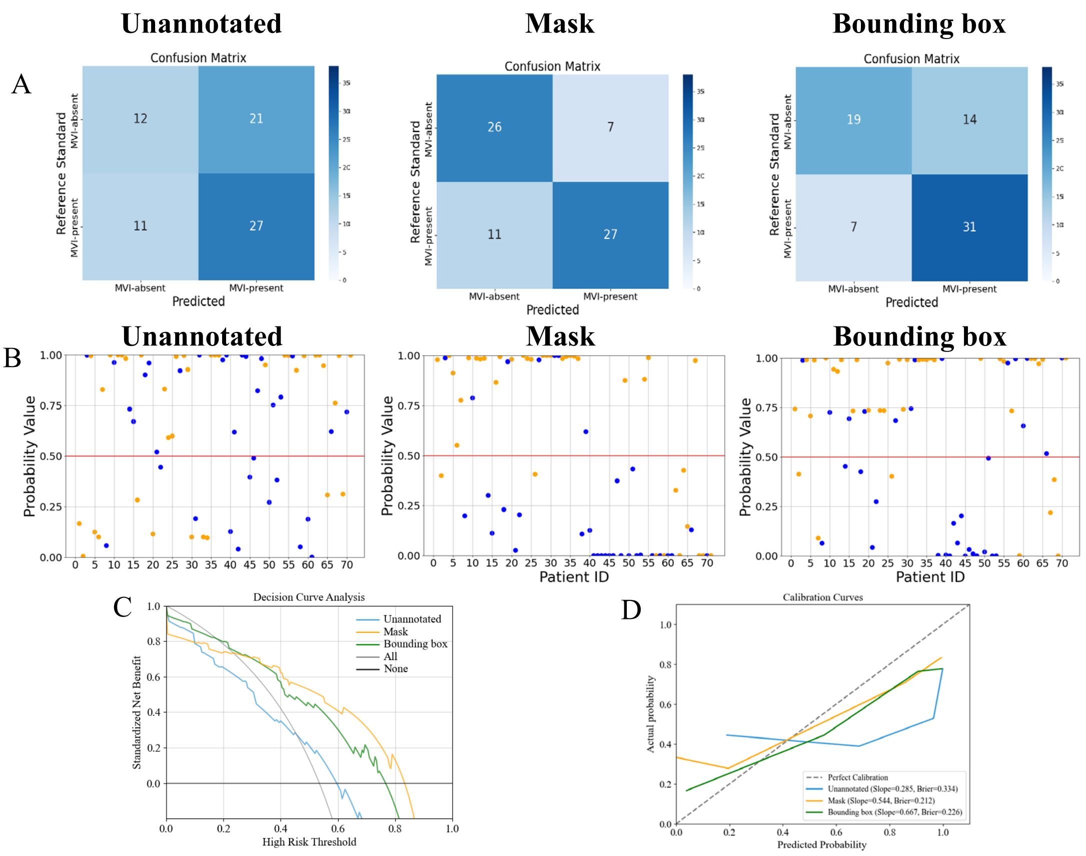

The (A) Confusion matrix diagrams, (B) probability scatter distribution diagrams, (C) decision curves and (D) calibration curves of the fusion models corresponding to the unannotated, mask, and bounding box in external test set.

Supplemental materials undergo peer review but are not copyedited, and may include typographical errors.

Figure S3. Visualization of Grad-CAM heatmaps for MVI-negative lesions generated by two annotation methods.

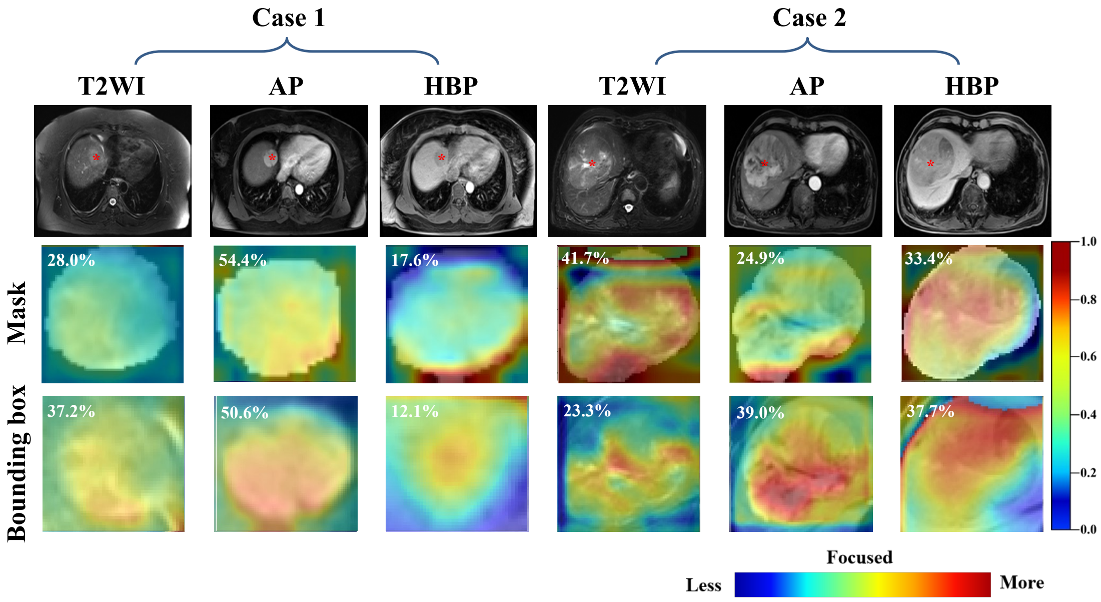

This figure shows T2-weighted imaging (T2WI), arterial phase (AP), and hepatobiliary phase (HBP) images along with heatmaps for two MVI-negative patients. The heatmaps represent two input types: (1) mask annotation, (2) bounding box annotation. The white numbers denote the Grad-CAM contribution proportions (percentage, %) of each sequence.

Supplemental materials undergo peer review but are not copyedited, and may include typographical errors.

Figure S4. Boxplot of Grad-CAM contribution proportion of different sequences under mask and bounding box annotation based models

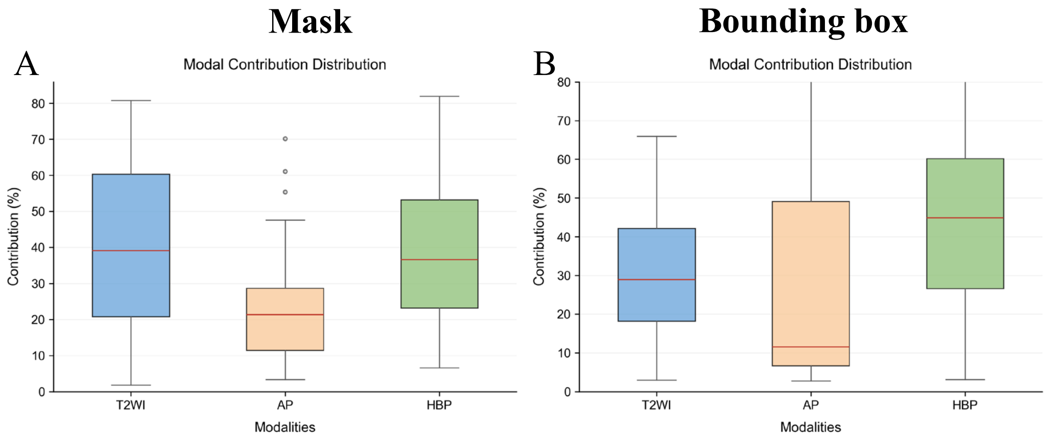

This figure illustrates the Grad-CAM contribution proportion (percentage, %) of the T2-weighted imaging (T2WI), arterial phase (AP), and hepatobiliary phase (HBP) sequences under two annotation based strategies: (A) mask annotation, and (B) bounding box annotation. Specifically, it displays boxplots that characterize the distribution of each modality’ s contribution across all samples.

Figure S5. Failure-Case of the Bounding Box Model

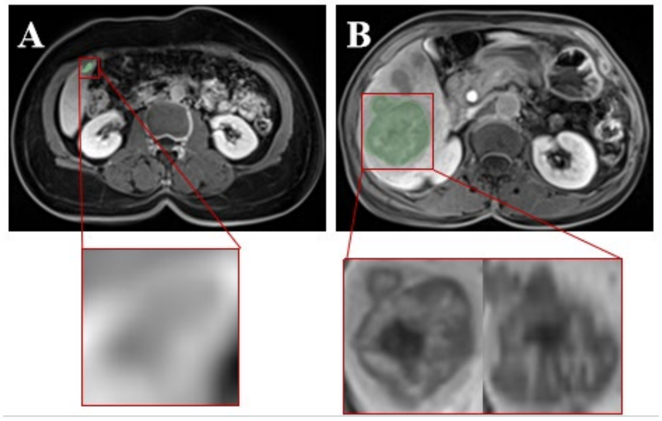

This figure shows hepatobiliary phase images of two typical HCC patients: (A) a 55-year-old female with MVI confirmed by postoperative pathology and a tumor measuring 1.2 cm in maximum diameter; (B) a 68-year-old female with MVI confirmed by postoperative pathology and an irregular tumor growth pattern.

Supplemental materials undergo peer review but are not copyedited, and may include typographical errors.

Appendix S1. MR protocol for center A, B and C

1) Center A: The patients included underwent upper abdominal MRI examination before surgery using a 3.0T MRI device (model: Magnetom Verio, Siemens Healthineers GmbH, Germany). Before scanning, the patient undergoes respiratory training using a supine and head first position, with an 8-channel phased array coil located at the center of the coil, 2-3 cm below the xiphoid process. The scanning protocol includes: (1) coronal T2WI imaging with half Fourier acquisition of a single shot turbo spin echo (Haste) sequence: repetition time (TR)/echo time (TE) of 1400/91 milliseconds, layer thickness of 5 mm, layer spacing of 1 mm, and a scanning field of view (FOV) of 36 × 36 cm^2; (2) Transversal diaphragmatic navigation fast spin echo (TSE) fat suppression T2WI sequence: TR/TE 2300/79 millisecond, layer thickness 5 mm, interlayer spacing 1 mm, FOV = 28 × 38 cm^2; (3) Transverse two-dimensional gradient echo fast small angle excitation T1WI sequence with same and opposite phases: TR/TE 133/1.23 milliseconds (opposite phase), 2.46 milliseconds (same phase), layer thickness of 5 mm, interlayer spacing of 1 mm, FOV 28 × 38 cm^2, flip angle (FA) 70 ° ; (4) Transverse DWI: b values of 50 and 800 sec/mm2, TR/TE 6000/73 milliseconds, layer thickness of 5 mm, interlayer spacing of 1 mm, FOV 28 × 38 cm^2, corresponding ADC images are automatically fitted and generated by the MRI post-processing system; (5) The cross-sectional T1WI volumetric interpolation breath hold examination (VIBE) sequence: TR/TE 3.9/1.4 millisecond, layer thickness 3 mm, interlayer spacing 0.6 mm, FOV = 28 × 38 cm^2, FA 9 ° . The contrast agent used was Gadobenate dimeglumine (Gd-BOPTA) injection. Before injecting the contrast agent, a plain T1WI-VIBE sequence image was collected as a pre scan for image correction. Then, a double tube high-pressure injector and a 19G internal trocar were used to inject the contrast agent through the median elbow vein at a dose of 0.1 mmol/kg and an injection rate of 2 ml/s. After the injection of contrast agent is completed, continue to inject 20 ml of physiological saline buffer at the same flow rate to flush out the residual contrast agent in the tube, while ensuring the effective concentration of contrast agent. AP, PVP, DP, and HBP

Supplemental materials undergo peer review but are not copyedited, and may include typographical errors.

images were collected at 20-25 seconds, 50-60 seconds, 2 minutes, and 60-90 minutes after injection of contrast agent.

2) Center B: All patients underwent preoperative upper abdominal MRI examinations using 3.0 T MRI scanners (VerioDot, Siemens Healthineers, Germany; uMR880, United Imaging Healthcare, Shanghai, China). The specific scanning protocols for VerioDot included:(1) Routine coronal T2-weighted imaging (T2WI) of the upper abdomen using a HASTE sequence with a matrix of 320 × 260 mm², field of view (FOV) of 400 × 360 mm², slice thickness of 6 mm, slice gap of 1.2 mm, repetition time/echo time (TR/TE) of 1400/114 ms;(2) Axial T2WI acquired by turbo spin-echo (TSE) sequence with a matrix of 320 × 320 mm², FOV 380 × 380 mm², slice thickness 5 mm, slice gap 1 mm, TR/TE 2500/87 ms;(3) Axial T1-weighted imaging (T1WI) using a breath-hold 3D-VIBE sequence, matrix 290 × 260 mm², FOV 380 × 310 mm², slice thickness 3 mm, TR/TE 4.5/2.05 ms, flip angle (FA) 16°.The scanning protocols for uMR880 included:(1) Routine coronal T2WI of the upper abdomen using fast spin-echo (FSE) sequence with a matrix of 320 ×260 mm², FOV 380 × 380 mm², slice thickness 6 mm, slice gap 2 mm, TR/TE 350/75 ms;(2) Axial T2WI using an ARMS sequence with FOV 380 × 380 mm², slice thickness 5 mm, slice gap 2 mm, TR/TE 3750/82 ms;(3) Axial T1WI acquired by a 3D volumetric interpolated fast gradient echo sequence with FOV 400 × 300 mm², slice thickness 3 mm, TR/TE 2.76/1.1 ms, FA 12°.After pre-contrast scans, all patients received manual intravenous injection of 10 mL Gadoxetic acid disodium (Gd-EOB-DTPA) via the antecubital vein, followed by dynamic contrast-enhanced scans. The injection was completed within 3–5 seconds, and images were acquired with a slice thickness of 3 mm at arterial phase (30 s), portal venous phase (60 s), delayed phase (3 min), and hepatobiliary phase (16 min) after contrast administration.

3) Center C: MRI was performed using a 3.0 T scanner (Achieva, Philips Healthcare, Netherlands) with a 16-channel phased-array coil. Routine MRI sequences included a free-breathing half-Fourier acquisition single-shot turbo spin-echo sequence, a breath-hold T1-weighted dual fast gradient recalled echo sequence (in-phase and

Supplemental materials undergo peer review but are not copyedited, and may include typographical errors.

opposed-phase), a breath-hold turbo spin-echo T2-weighted fast spin-echo sequence with fat suppression, and free-breathing diffusion-weighted imaging with low and high b-values (b = 0, 800 s/mm², respectively). Dynamic contrast-enhanced MRI was conducted using a fat-suppressed T1-weighted three-dimensional gradient echo sequence (time of repetition/time of echo (TR/TE) = 4.0–4.5 / 1.5–2.0 ms, flip angle 15°, matrix 180×320, slice thickness 5.0–6.0 mm, field of view 440 × 440 mm). After intravenous injection of 0.1–0.15 mmol/kg contrast agent (Gadobenate Dimeglumine, Gd- BOPTA) at a rate of 2.5 mL/s, arterial phase (25 s), portal venous phase (60 s), and delayed phase (3 min) images were acquired. Hepatobiliary phase images were obtained 1 hour after injection.

Supplemental materials undergo peer review but are not copyedited, and may include typographical errors.

Table S1: Clinical variables of training and internal test sets

Clinical variables	Overall (n=210)	Training set (n = 147)			Internal test set (n = 63)			P_{inter}
		MVI+ (n = 76)	MVI− (n = 71)	P_{intra}	MVI+ (n = 33)	MVI− (n = 30)	P_{intra}	
Sex				.69			.17	.15
Female	32 (15.2)	9 (11.8)	10 (14.1)		9 (27.3)	4 (13.3)		
Male	178 (84.8)	67 (88.2)	61 (85.9)		24 (72.7)	26 (86.7)		
Age (y, mean ± SD)	59.0±11.7	60.3±11.7	59.8±10.9	.80	54.6±12.3	58.5±12.9	.14	.10
Range	15,85	27,81	33,85		29,74	15,75		
Number of tumors				.89			>.99	.84
Solitary	188 (89.5)	68 (89.5)	64 (90.1)		29 (87.9)	27 (90.0)		
Multiple	22 (10.5)	8 (10.55)	7 (9.9)		4 (12.1)	3 (10.0)		
Cirrhosis				.77			.41	.32
Absent	76 (36.2)	25 (32.9)	25 (35.2)		12 (36.7)	14 (46.7)		
Present	134 (63.8)	51 (67.1)	46 (64.8)		21 (63.6)	16 (53.3)		
HBV/HCV				.27			.84	.44
Negative	40 (19.0)	16(21.1)	10(14.1)		7 (21.2)	7 (23.3)		
Positive	170 (81.0)	60(78.9)	61(85.9)		26 (78.8)	23 (76.7)		
AFP				.001			.01	.54
≤ 400 ng/ml	156 (74.3)	49 (64.5)	62(87.3)		19 (57.6)	26 (21.3)		
> 400 ng/ml	54 (25.7)	27(35.5)	9(12.7)		14 (42.4)	4 (86.7)		
PLT				.10			>.99	.35
≤ 125×10^9/L	38 (18.1)	19(25.0)	10(14.1)		5 (15.2)	4 (13.3)		
> 125×10^9/L	172 (81.9)	57(75.0)	61(85.9)		28 (84.8)	26 (25.7)		
PT				.56			.78	.82
≤ 13 s	139 (66.2)	49(64.5)	49(69.0)		22 (66.7)	19 (63.3)		
> 13 s	71 (33.8)	27(35.5)	22(31.0)		11 (33.3)	11 (36.7)		
PT-INR				.42			.93	.54
≤ 1.0	56 (26.7)	19(25.0)	22(31.0)		8 (24.2)	7 (23.3)		
> 1.0	154 (73.3)	57(75.0)	49(69.0)		25 (75.8)	23 (76.7)		
TBIL				.29			.13	.30

Supplemental materials undergo peer review but are not copyedited, and may include typographical errors.

≤ 20.5 μmol/L	166 (79.0)	59 (77.6)	60 (84.5)		22 (66.7)	25 (83.3)		
> 20.5 μmol/L	44 (21.0)	17 (22.4)	11 (15.5)		11 (33.3)	5 (16.7)		
ALB				>.99			.80	.54
≤ 40 g/L	80 (38.1)	30 (39.5)	28 (39.4)		12 (36.4)	10 (33.3)		
> 40 g/L	130 (61.9)	46 (60.5)	43 (60.6)		21 (63.6)	20 (66.7)		
GGT				.27			.36	.10
≤ 60 U/L	131 (62.4)	47 (61.8)	50 (70.4)		16 (48.5)	18 (60.0)		
> 60 U/L	79 (37.6)	29 (38.2)	21 (29.6)		17 (51.5)	12 (40.0)		
ALT				.68			.31	.37
≤ 50 U/L	171 (81.4)	64 (84.2)	58 (81.7)		24 (72.7)	25 (83.3)		
> 50 U/L	39 (18.6)	12 (15.8)	13 (18.3)		9 (27.3)	5 (16.7)		
AST				.68			.08	0.24
≤ 40 U/L	164 (78.1)	60 (79.0)	58 (81.7)		21 (63.6)	25 (83.3)		
> 40 U/L	46 (21.9)	16 (21.0)	13 (18.3)		12 (36.4)	5 (16.7)		

HBV/HCV = hepatitis B or C virus infection, AFP = α -fetoprotein, ALT = alanine aminotransferase, AST = aspartate aminotransferase, PLT = platelet count, PT = prothrombin time, PT-INR = prothrombin time - international normalized ratio, TBIL = total bilirubin, ALB = albumin, GGT = gamma-glutamyltransferase. Except for age, which is expressed as mean and standard deviation, all other data are patient numbers, with percentages in parentheses. *P* value with Chi-square test or Fisher exact test for categorical variables. P_{intra}: p value between the MVI+ and MVI− groups in the training and test cohort. P_{inter}: *P* value between the training and test cohorts.

Supplemental materials undergo peer review but are not copyedited, and may include typographical errors.

Table S2: Radiological variables of training and internal test sets

Radiological variables	Overall (n=210)	Training set (n = 147) MVI+ (n = 76)	MVI− (n = 71)	P_{intra}	Internal test set (n = 63) MVI+ (n = 33)	MVI− (n = 30)	P_{intra}	P_{inter}
Maximum tumor length				.06			<.001	.11
≤ 5cm	121 (57.6)	41 (54.0)	49 (69.0)		8 (24.2)	23 (76.7)		
> 5cm	89 (42.4)	35 (46.0)	22 (31.0)		25 (75.8)	7 (23.3)		
Tumor margins				<.001			.06	.45
Smooth	95 (45.2)	20 (26.3)	49 (69.0)		10 (30.3)	16 (53.3)		
Non-smooth	115 (54.8)	56 (73.7)	22 (31.0)		23 (69.7)	14 (46.7)		
APHE				.004			.02	.39
Absent	33 (15.7)	17 (22.4)	4 (5.6)		10 (30.3)	2 (6.7)		
Present	177 (84.3)	59 (77.6)	67 (94.4)		23 (69.7)	28 (93.3)		
Nonperipheral washout				.01			.28	.70
Absent	53 (25.2)	12 (15.8)	24 (33.8)		7 (21.2)	10 (33.3)		
Present	157 (74.8)	64 (84.2)	47 (66.2)		26 (78.8)	20 (66.7)		
Peritumoral_ arterial enhancement				.22			.02	.08
Absent	127 (60.5)	45 (59.2)	49 (69.0)		12 (36.4)	20 (66.7)		
Present	83 (39.5)	31 (40.8)	22 (31.0)		21 (63.6)	10 (33.3)		
Tumor capsule				.02			.41	.36
Complete	90 (42.9)	27 (35.5)	39 (54.9)		11 (33.3)	13 (43.3)		
Absent/Incomplete	120 (57.1)	49 (64.5)	32 (45.1)		22 (66.7)	17 (56.7)		
Tumor hypointensity on HBP				.35			.60	.43
Absent	7 (3.3)	1 (1.3)	3 (4.2)		1 (3.0)	2 (6.7)		
Present	203 (96.7)	75 (98.7)	68 (95.8)		32 (97.0)	28 (93.3)		
Peritumoral hypointensity on HBP				<.001			.01	.09
Absent	144 (68.6)	44 (57.9)	62 (87.3)		15 (45.5)	23 (76.7)		
Present	66 (31.4)	32 (42.1)	9 (12.7)		18 (54.5)	7 (23.3)		
Mosaic architecture				.007			.02	.25
Absent	86 (41.0)	25 (32.9)	39 (54.9)		7 (21.2)	15 (50.0)		
Present	124 (59.0)	51 (67.1)	32 (45.1)		26 (78.8)	15 (50.0)		

Supplemental materials undergo peer review but are not copyedited, and may include typographical errors.

Hemorrhage				.14			.007	.23
Absent	152 (72.4)	53 (69.7)	57 (80.3)		17 (51.5)	25 (83.3)		
Present	58 (27.6)	23 (30.3)	14 (19.7)		16 (48.5)	5 (16.7)		
Necrosis				.01			.004	.55
Absent	159 (75.7)	52 (68.4)	61 (85.9)		19 (57.6)	27 (90.0)		
Present	51 (24.3)	24 (31.6)	10 (14.1)		14 (42.4)	3 (10.0)		
Steatosis				.29			.29	.54
Absent	175 (83.3)	65 (85.5)	56 (78.9)		30 (90.9)	24 (80.0)		
Present	35 (16.7)	11 (14.5)	15 (21.1)		3 (9.1)	6 (20.0)		

Data are numbers of patients, with the percentage in parentheses. APHE = arterial phase hyperenhancement; HBP = hepatobiliary phase. *P* value with Chi-square test or Fisher exact test for categorical variables. P_{intra}: *P* value between the MVI+ and MVI− groups in the training and test cohort. P_{inter}: *P* value between the training and test cohorts.

Supplemental materials undergo peer review but are not copyedited, and may include typographical errors.

Table S3: Clinical variables and radiological variables of Center B (n = 40)

Clinical variables	MVI+ (n = 24)	MVI− (n = 16)	Radiological variables	MVI+ (n = 24)	MVI− (n = 16)
Sex			Tumor margins		
Female	4 (16.7)	3 (18.7)	Smooth	7 (29.2)	12 (75.0)
Male	20 (83.3)	13 (81.3)	Non-smooth	17 (70.8)	4 (25.0)
Age(y, mean ± SD)	57.8±14.3	60.1±13.3	APHE		
HBV/HCV			Absent	4 (16.7)	2 (12.5)
Negative	3 (12.5)	2 (12.5)	Present	20 (83.3)	14 (87.5)
Positive	21 (87.5)	14 (87.5)	Nonperipheral washout		
AFP			Absent	2 (8.3)	1 (6.3)
≤ 400 ng/ml	16 (66.7)	13 (81.2)	Present	22 (91.7)	15 (93.7)
> 400 ng/ml	8 (33.3)	3 (18.8)	Peritumoral_ arterial enhancement		
TBIL			Absent	20 (83.3)	16 (100)
≤ 20.5 μmol/L	22 (83.3)	15 (93.7)	Present	4 (16.7)	0 (0.0)
> 20.5 μmol/L	11 (16.7)	1 (6.3)	Tumor capsule		
GGT			Complete	20 (45.8)	8 (50.0)
≤ 60 U/L	14 (58.3)	14 (87.5)	Absent/Incomplete	4 (54.2)	8 (50.0)
> 60 U/L	10 (41.7)	2 (12.5)	Tumor hypointensity on HBP		
ALT			Absent	0 (0.0)	0 (0.0)
≤ 50 U/L	18 (75.0)	13 (81.3)	Present	24 (100)	16 (100)
> 50 U/L	6 (25.0)	3 (18.7)	Peritumoral hypointensity on HBP		
AST			Absent	21 (87.5)	15 (93.7)
≤ 40 U/L	17 (70.8)	14 (87.5)	Present	3 (12.5)	1 (6.3)
> 40 U/L	7 (29.2)	2 (12.5)			

HBV/HCV = hepatitis B or C virus infection, AFP = α -fetoprotein, ALT = alanine aminotransferase, AST = aspartate aminotransferase, TBIL = total bilirubin, GGT = gamma-glutamyltransferase, SD = standard deviation, APHE = arterial phase hyperenhancement; HBP = hepatobiliary phase. Except for age, which is expressed as mean and standard deviation, all other data are patient numbers, with percentages in parentheses.

Supplemental materials undergo peer review but are not copyedited, and may include typographical errors.

Table S4: Clinical variables and radiological variables of Center C (n = 31)

Clinical variables	MVI+ (n = 13)	MVI− (n = 18)	Radiological variables	MVI+ (n = 13)	MVI− (n = 18)
Sex			Tumor margins		
Female	0 (0)	4 (22.2)	Smooth	1 (7.7)	14 (77.8)
Male	13 (100)	14 (77.8)	Non-smooth	12 (92.3)	4 (22.2)
Age(y, mean ± SD)	60.2±10.2	59.5±11.3	Nonperipheral washout		
AFP			Absent	3 (23.1)	7 (38.9)
≤ 400 ng/ml	9 (69.2)	15 (83.3)	Present	10 (76.9)	11 (61.1)
> 400 ng/ml	4 (30.8)	3 (16.7)	Peritumoral hypointensity on HBP		
			Absent	8 (61.5)	17 (94.4)
			Present	5 (38.5)	1 (5.6)

AFP = α-fetoprotein, SD = standard deviation; HBP = hepatobiliary phase. Except for age, which is expressed as mean and standard deviation, all other data are patient numbers, with percentages in parentheses.

Table S5: The performance of fusion models with different degrees of annotation in external test set

Model	AUC (95% CI)	Accuracy (%)	Sensitivity (%)	Specificity (%)
Unannotated	0.61 (0.48,0.75)	54.9(39/71)	71.1(27/38)	36.4(12/33)
Mask	0.77 (0.66,0.89)	74.7(53/71)	71.1(27/38)	78.8(26/33)
Bounding box	0.76 (0.64,0.88)	70.4(50/71)	81.6(31/38)	57.6(19/33)

AUC = area under the receiver operating characteristic curve; CI = confidence interval. Accuracy, sensitivity, and specificity are presented as percentages (number of correct cases/total cases).

Table S6: Proportion of L_2 norm in each branch of fusion models with different degrees of annotation in internal test set

Model	Unannotated				Mask				Bounding box			
**Branch**	T2WI	AP	HBP	C-R	T2WI	AP	HBP	C-R	T2WI	AP	HBP	C-R
**Mean ± SD**	0.22 ± 0.13	0.29 ± 0.15	0.45 ± 0.17	0.04 ± 0.03	0.23 ± 0.11	0.25 ± 0.11	0.48 ± 0.18	0.05 ± 0.03	0.24 ± 0.12	0.28 ± 0.16	0.41 ± 0.14	0.06 ± 0.06
**Weight(%)**	21.97	29.02	45.36	3.65	23.28	24.63	47.57	4.52	28.36	24.45	41.14	6.05

T2WI = T2-weighted imaging, AP = arterial phase, HBP = hepatobiliary phase, C-R = clinical-radiological branch, SD = standard deviation. Weight (%) = proportion of L2 norm contribution of each branch to the fusion model.

Supplemental materials undergo peer review but are not copyedited, and may include typographical errors.

Table S7: Pairwise DeLong test results for model comparisons across test sets

Model Type	test set	Delong Test	
		AUC value	*P* value
Unannotated vs Mask	Internal	0.73 vs 0.88	.04
	External	0.61 vs 0.77	.02
Unannotated vs Bounding box	Internal	0.73 vs 0.85	.03
	External	0.61 vs 0.76	.04
Mask vs Bounding box	Internal	0.88 vs 0.85	.50
	External	0.77 vs 0.76	.40

AUC = area under the receiver operating characteristic curve, *P* values were calculated using the DeLong test for pairwise comparisons of ROC curves.

Table S8:Comparison of Grad-CAM contribution proportion of different sequences between two methods in internal test set

Sequence	Mask	Bounding box
T2WI	39.22 ± 23.27	30.06 ± 17.35
AP	22.11 ± 14.30	27.66 ± 28.29
HBP	38.67 ± 19.86	42.28 ± 21.16

T2WI = T2-weighted imaging, AP = arterial phase, HBP = hepatobiliary phase, SD = standard deviation. All values in the table represent contribution percentages (%).

Table S9: Comparison of time used for two annotation methods

Annotation method	Average time (minutes/lesion)	Standard deviation (minutes)	Time range (minutes)
Mask	3.24	1.18	1.25 - 6.81
Bounding box	1.52	0.32	1.00 - 2.67

Time is measured as the duration required to annotate one lesion, presented as minutes (min).
